# Supplementary material for: Topographic gradients of intrinsic dynamics across neocortex
Source: eLife. 2020 Dec 17;9:e62116. doi: 10.7554/eLife.62116 (PMC7771969; doi:10.7554/eLife.62116)
Supplement: Supplementary file 1. — Dominance Analysis was used to quantify the distinct contributions of inter-regional Euclidean distance, structural connectivity, and functional connectivity to temporal profile similarity (Budescu, 1993; Azen and Budescu, 2003) (https://github.com/dominance-analysis/dominance-analysis). Dominance analysis is a method for assessing the relative importance of predictors in regression or classification models. The technique estimates the relative importance of predictors by constructing all possible combinations of predictors and quantifying the relative contribution of each predictor as additional variance explained (i.e. gain in R2) by adding that predictor to the models. Specifically, for p predictors we have 2p-1 models that include all possible combinations of predictors. The incremental R2 contribution of each predictor to a given subset model of all the other predictors is then calculated as the increase in R2 due to the addition of that predictor to the regression model. Here we first constructed a multiple linear regression model with distance, structural connectivity and functional connectivity as independent variables and temporal profile similarity as the dependent variable to quantify the distinct contribution of each factor using dominance analysis. The total R2 is 0.28 for the complete model that includes all variables. The relative importance of each factor is summarized in the table, where each column corresponds to: Interactional Dominance is the incremental R2 contribution of the predictor to the complete model. For each variable, interactional dominance is measured as the difference between the R2 of the complete model and the R2 of the model with all other variables except that variable; Individual Dominance of a predictor is the R2 of the model when only that predictor is included as the independent variable in the regression; Average Partial Dominance is the average incremental R2 contributions of a given predictor to all possible subset of mode [file elife-62116-supp1.docx]

|  | Interactional Dominance | Individual Dominance | Average Partial Dominance | Total Dominance | Percentage Relative Importance |
| --- | --- | --- | --- | --- | --- |
| Euclidean distance | 0.095 | 0.245 | 0.128 | 0.156 | 56.015 |
| functional connectivity | 0.030 | 0.130 | 0.038 | 0.066 | 23.591 |
| structural connectivity | 2.967e-05 | 0.142 | 0.029 | 0.057 | 20.393 |

**Supplementary File 1. Dominance analysis |** Dominance Analysis was used to quantify the distinct contributions of inter-regional distance, structural connectivity, and functional connectivity to temporal profile similarity (Budescu, 1993; Azen & Budescu, 2003; <https://github.com/dominance-analysis/dominance-analysis>). Dominance analysis is a method for assessing the relative importance of predictors in regression or classification models. The technique estimates the relative importance of predictors by constructing all possible combinations of predictors and quantifying the relative contribution of each predictor as additional variance explained (i.e. gain in R^2^) by adding that predictor to the models. Specifically, for *p* predictors we have 2*^p^*-1 models that include all possible combinations of predictors. The incremental R^2^ contribution of each predictor to a given subset model of all the other predictors is then calculated as the increase in R^2^ due to the addition of that predictor to the regression model. Here we first constructed a multiple linear regression model with distance, structural connectivity and functional connectivity as independent variables and temporal profile similarity as the dependent variable to quantify the distinct contribution of each factor using dominance analysis. The total R^2^ is 0.28 for the complete model that includes all variables. The relative importance of each factor is summarized in the table, where each column corresponds to: Interactional Dominance is the incremental R^2^ contribution of the predictor to the complete model. For each variable, interactional dominance is measured as the difference between the R^2^ of the complete model and the R^2^ of the model with all other variables except that variable; Individual Dominance of a predictor is the R^2^ of the model when only that predictor is included as the independent variable in the regression; Average Partial Dominance is the average incremental R^2^ contributions of a given predictor to all possible subset of models except the complete model and the model that only includes that variable; Total Dominance is a summary measure that quantifies the additional contribution of each predictor to all subset models by averaging all the above measures for that predictor; Percentage Relative Importance is the percent value of the Total Dominance.
