# Supplementary material for: Topographic gradients of intrinsic dynamics across neocortex
Source: eLife. 2020 Dec 17;9:e62116. doi: 10.7554/eLife.62116 (PMC7771969; doi:10.7554/eLife.62116)
Supplement: Supplementary file 2. — The overlapping terms between Neurosynth (Yarkoni et al., 2011) and Cognitive Atlas (Poldrack et al., 2011) corpuses used in the reported analyses are listed below. [file elife-62116-supp2.docx]

| action | eating | insight | naming | semantic memory |
| --- | --- | --- | --- | --- |
| adaptation | efficiency | integration | navigation | sentence comprehension |
| addiction | effort | intelligence | object recognition | skill |
| anticipation | emotion | intention | pain | sleep |
| anxiety | emotion regulation | interference | perception | social cognition |
| arousal | empathy | judgment | planning | spatial attention |
| association | encoding | knowledge | priming | speech perception |
| attention | episodic memory | language | psychosis | speech production |
| autobiographical memory | expectancy | language comprehension | reading | strategy |
| balance | expertise | learning | reasoning | strength |
| belief | extinction | listening | recall | stress |
| categorization | face recognition | localization | recognition | sustained attention |
| cognitive control | facial expression | loss | rehearsal | task difficulty |
| communication | familiarity | maintenance | reinforcement learning | thought |
| competition | fear | manipulation | response inhibition | uncertainty |
| concept | fixation | meaning | response selection | updating |
| consciousness | focus | memory | retention | utility |
| consolidation | gaze | Memory retrieval | retrieval | valence |
| context | goal | mental imagery | reward anticipation | verbal fluency |
| coordination | hyperactivity | monitoring | rhythm | visual attention |
| decision | imagery | mood | risk | visual perception |
| decision making | impulsivity | morphology | rule | word recognition |
| detection | induction | motor control | salience | working memory |
| discrimination | inference | movement | search |  |
| distraction | inhibition | multisensory | selective attention |  |

**Supplementary File 2. List of terms used in Neurosynth analyses |** The overlapping terms between Neurosynth (Yarkoni et al., 2011) and Cognitive Atlas (Poldrack et al., 2011) corpuses used in the reported analyses are listed below.
